# Participant selection procedures in qualitative research: experiences and some points for consideration

**DOI:** 10.3389/frma.2024.1512747

**Published:** 2024-12-12

**Authors:** Niroj Dahal, Bharat Prasad Neupane, Binod Prasad Pant, Rebat Kumar Dhakal, Dhudi Raj Giri, Puna Ram Ghimire, Laxman Prasad Bhandari

**Affiliations:** ^1^Kathmandu University School of Education, Lalitpur, Nepal; ^2^Tribhuvan University, Kirtipur, Nepal

**Keywords:** participant selection, auto/ethnography, narrative inquiry, participatory action research, ethnography, case study, grounded theory, phenomenology

## Abstract

Qualitative research is widely embraced in the social sciences and education. Among the different traditional, modern, and community-oriented qualitative methodologies, we have drawn on our experiences to adopt seven qualitative methodologies: auto/ethnography, narrative inquiry, participatory action research, ethnography, case study, grounded theory, and phenomenology. Despite the abundance of literature on qualitative methodologies, there is still a need for a more focused exploration of participant selection procedures in qualitative studies. This article examines the discourse around participant selection procedures within these seven methodologies, highlighting their unique nuances and differences. It offers practical insights and guidelines for novice and experienced researchers and graduate and postgraduate students to enhance their understanding of participant selection procedures, and some thinking points for consideration. Drawing from our experiences, we aim to provide a useful resource that encourages thoughtful consideration of participant selection in qualitative studies.

## 1 Introduction

In this article, we report our experiences of participant selection in different traditional, modern, and community-oriented qualitative methodologies—auto/ethnography, narrative inquiry, participatory action research, ethnography, case study, grounded theory, and phenomenology, and some thinking points for consideration. Qualitative research is gaining popularity in social science and educational research for exploring human experiences and feelings. Humans aligned to the phenomenon are the main information and/or data sources. Aspers and Corte (2019) noted that this popularity should be “Seen in a historical light, what is today called qualitative, or sometimes ethnographic, interpretative research—or a number of other terms—has more or less always existed.” (p. 141). Denzin and Lincoln (2002) stated, “Qualitative research is a situated activity that locates the observer in the world. It consists of a set of interpretive, material practices that make the world visible. These practices transform the world. They turn the world into a series of representations, including field notes, interviews, conversations, photographs, recordings, and memos to the self.” (p. 3). Thus, qualitative research plays a crucial role in exploring complex phenomena and gaining in-depth insights into human experiences. Further, Pyo et al. (2023) remarked that “Qualitative research is conducted in the following order: (1) selection of a research topic and question, (2) selection of a theoretical framework and methods, (3) literature analysis, (4) selection of the research participants and data collection methods, (5) data analysis and description of findings, and (6) research validation.” (p. 12). However, there is always back and forth between different processes, making it iterative. Despite the popularity of qualitative research, novice researchers often struggle with the intricacies of participant selection for exploring human experiences aligned with their feelings, emotions, and perceptions. So, selecting the appropriate research participants is crucial to conducting any qualitative research and/or inquiry that directly influences the rigor—credibility, and richness of the data collection and/or generation. However, inappropriate choice of participants and data collection may lead to methodological flaws and compromised study outcomes.

With advantages, disadvantages, and characteristics, the participant selection procedure in qualitative research is considered purposeful sampling with referred criteria in general and co-researchers in particular. These selection procedures are often based on problem, purpose, research question, and theoretical referents. In this article, we have explored participants' selection procedures, drawings from our experiences and understanding. We offer some thinking points for consideration in qualitative methods and the nuanced differences and uniqueness in each of the chosen methodologies by offering a practical guide for consideration for novice and/or veteran researchers regarding participant selection in seven qualitative methodologies—auto/ethnography, narrative inquiry, participatory action research, ethnography, case study, phenomenology, and grounded theory.

Choosing appropriate participant selection procedures is essential to enhance the quality of qualitative studies. This paper serves as a comprehensive guide for novice and/or veteran researchers, offering a step-by-step approach to participant selection in the chosen qualitative research method, taking care of challenges, and offering practical solutions based on our studies. In this article, we report our experiences of participant selection in each methodological tradition, as the process is essential for ensuring quality in qualitative research findings and/or outcomes (Dahal, 2023). Addressing the ethical considerations and practical tips for selecting participants, this article offers a participant selection procedure in seven qualitative methodologies (1) auto/ethnography, (2) narrative inquiry, (3) participatory action research, (4) ethnographic study, (5) case study, (6) grounded theory, and (7) phenomenology. As the authors have also embedded reflective experiences and some thinking points for consideration of our research journey in relation to participant selection, we have used the first-person pronoun “I” in subsequent sections.

## 2 Experiences and some points for consideration 1: auto/ethnographic inquiry

In this section, I, the first and corresponding author, share my experiences of conducting auto/ethnographic inquiry, particularly the participant selection process. In my auto/ethnographic inquiry, the research site was not confined to boundaries because of its unique nature. Based on my research purpose, my inquiry was confined to myself, four research participants, and a critical friend based in the South Asian context. This limitation, however, suggested exploring the information through postmodern approaches (Gubrium and Holstein, 2003) of *self* and *others* connecting life and research (Dahal et al., 2022). Postmodern approaches encouraged me to uncover my beliefs, thinking, and process of being and becoming in a larger context, thinking qualitatively. Likewise, the main motto of my auto/ethnographic inquiry was to explore anecdotal and personal experiences of self (insider) and others (culture) and connect the autobiographical story to wider cultural and social meanings and understandings to enrich the meaning-making process from my research participants—Aarati, Kamal, Hari, and Santosh, and my critical friend—Naresh. However, the names and institutions they serve are pseudonyms.

Next, in my PhD inquiry (Dahal, 2024), I primarily generated data from myself by incorporating the research participants and critical observations from a critical friend. My conversations were followed based on the narrative generated by myself with four research participants and a critical friend observation while envisioning the science, technology, engineering, the arts, and mathematics (STEAM)-based mathematics education on (1) school-community relations, (2) mathematical curricular spaces, (3) professional development, and (4) leadership development in STEAM-based mathematics education (Gubrium and Holstein, 2003). In contrast, I was flexible in terms of the number of research participants: first six, then five, and four. These were the basic tenets of my auto/ethnography as per the emerging nature of the inquiry. Data generation was carried out before, during, and after the field engagements. I employed writing as/for the process of the inquiry to capture the contextual and universal perspectives in my inquiry. Data generation by incorporating the research participants and critical observations from the critical friend took almost 2 years (2021–2023) and other professional engagements while continuously envisioning the STEAM-based mathematics education, even in the finalizing stage of the thesis. In my PhD study, I have presented a brief description of my participants as follows:

**Aarati** is a secondary-level mathematics teacher in one of the private schools in Kathmandu Valley, Nepal. She is from the rural part of Nepal. She has been involved in the teaching profession after completing her M Ed in Mathematics education in the year 2014 from one of the reputed universities of Nepal. She knows the pedagogical approaches of teaching and learning mathematics. But she does take care of other engagements to be professional, such as attending professional training and conducting the training in school.

**Kamal** has been in the teaching profession for more than three decades. He completed his master's degree in mathematics education in 2012. After completing a master's degree, he has been engaging in the teaching profession. He has also been engaged as a seasonal teacher trainer to facilitate professional training for mathematics teachers in different parts of Nepal. He is the head of the mathematics department in his school in Lalitpur, Nepal.

**Hari** has completed his MPhil degree at a reputed university in Nepal. He has been in the teaching profession after completing a B Ed in Mathematics education in the year 2009. He is a permanent secondary-level mathematics teacher in one of the government schools in Lalitpur, Nepal. He has gained experience in teaching and learning by incorporating innovation. Likewise, he is also leading the mathematics department in the school. He has not limited his engagement to teaching and learning mathematics in school, but he has also been involved in offering professional training in different schools.

**Santosh** works as a secondary-level mathematics teacher at one of the government schools in Bhaktapur, Nepal. He completed B. Ed., then M. Ed., and MPhil. in Mathematics education. He completed a B. Ed. in 2010. Besides being a secondary-level mathematics teacher, he has been a part-time lecturer at one of the Tribhuvan University, Nepal campuses. In school, he also served as the school's training head to facilitate the schoolteachers' professional development training. Offering ample opportunity for the schoolteachers is one of his dreams to achieve in his profession. He seems like a passionate mathematics teacher who attempts to blend different strategies for school overall development by blending local and global knowledge in mathematics and wisdom.

**Naresh** is from remote part of Nepal and struggled a lot in his past studies—SLC (now named SEE), intermediate, and Bachelor. He completed his master's degree in mathematics education in 2018. After master's degree, he engaged in private and public institutions to uplift his career in various positions. Continuing his studies, he also completed his MPhil in STEAM education in 2021 at one of the universities in Nepal. Likewise, he has been teaching various university-level courses. He has also presented some scholarly works at national and international conferences. His research interests are STEAM education and ICT and mathematics education. He believes in reflective practices to transform the meaningful engagement of the learners.

Thus, a brief description of participants in the method section of the article and/or thesis makes the research method more transparent as participant selection procedures.

## 3 Experiences and some points for consideration 2: narrative inquiry

In this section, I, the second author, briefly discuss the narrative inquiry and then the participant selection process in detail. Narrative inquiry is an emerging research method that is gaining popularity in social science research in general and educational and teacher education research in particular. Narrative inquiry mainly predominates research studies exploring “educational experience as lived” (Connelly and Clandinin, 1990, p. 3). In narrative research, we explore participants' lived experiences that are recorded in the form of stories.

Highlighting the importance of stories in human life, Silko (2006) mentions, “You don't have anything if you don't have stories” in her widely acknowledged novel *Ceremony*. Human beings are storytelling creatures who explain their and others' doings through narratives of past, present, and imagined future experiences. According to Kramp (2004), stories “assist humans to make life experiences meaningful. Stories preserve our memories, prompt our reflections, connect us to our past and present, and assist us to envision our future” (p. 107). Barkhuizen (2016) argues that “Experiences become narratives when we tell them to an audience, and narratives become part of narrative inquiry when they are examined for research purposes or generated to report the findings of an inquiry” (p. 4). According to Webster and Mertova (2007), narratives allow teachers and researchers to present experiences holistically with their complex situatedness. Narrative inquiry is concerned with analyzing, interpreting, critiquing, and presenting stories we live by, be it individual storied life or myths surrounding us. The critique process means that the research process can indeed become a matter of co-generating imagined future experiences (Silko, 2006). As people live storied lives and narrate the stories of such lives, the primary responsibility of the researcher in narrative inquiry is to present such lived experiences with their meaning, during which even the researcher becomes part of the meaning-making process where they construct and reconstruct a shared narrative through inquiry (Barkhuizen et al., 2014; Connelly and Clandinin, 1990).

Narratives occur in specific socio-cultural contexts with three main commonplaces: temporality, sociality, and place, constituting the concept of narrative inquiry. These commonplaces, always in the process of becoming or transitioning, distinguish narrative inquiry from other methodologies (Clandinin and Huber, 2010; Connelly and Clandinin, 1990; Clandinin et al., 2007). Contextual factors embedded in participants' narratives make narrative data rich and complex. As Connelly and Clandinin (1990) noted, data collection methods ranging from field notes of the shared experience to in-depth interviews, journals, and other sources make it rich. Most often, narrative inquiry involves a minimal number of research participants (Riessman, 1993), sometimes just one, primarily in the case of the life history approach, with an in-depth and prolonged period of story generation (Polkinghorne, 1995). The type of narrative inquiry—autobiographical, biographical, life history, arts-based—and the data collection method chosen by the researcher also influences the number of research participants. For instance, if a researcher is willing to conduct the narrative survey first and then select a chosen few as research participants, 30 participants are generally considered an ideal number. However, for in-depth interviews to explore the rich data, six to ten participants suffice the purpose of the research.

In the case of the autobiographical and life history approach, even just one participant is enough. The prolonged engagement is crucial in the life history approach of narrative inquiry instead of numbers. In my PhD study that explored the trajectory of identity negotiation of English language teachers in Nepal subscribing to the life history approach, I have taken just four secondary-level English language teachers in public schools in Kathmandu Valley as my research participants. Like other qualitative research approaches, even in narrative inquiry, purposive sampling is relevant in selecting participants based on the criteria. In my research studies (Neupane and Bhatt, 2023; Neupane, 2024), the participant's selection criteria were defined as (a) teachers having at least one education degree, either Master of Education (M.Ed.) or Master of Philosophy (MPhil), (b) currently teaching secondary-level students in public schools in Nepal, and (c) having at least seven to 10 years of teaching experience. However, before deciding on the four participants who met my criteria, I had a primary level of conversation with seven participants. Out of those seven participants, four participants who best fit the defined criteria were selected. Experienced teachers were purposively selected as research participants as they could achieve a certain level of maturity, gain ample experience, and attain a certain level of identity construction during this stage.

In narrative inquiry, participant selection is primarily purposive, and the selection is done based on defined criteria to meet the purpose of the study. Next, the type of method that we have chosen also influences the number of participants. In narrative research, prolonged engagement and drawing detailed experiences are crucial instead of the number of participants. In narrative research, researchers can also imbed their experiences as data. In my PhD research and participants' stories, I included my lived experiences wherever relevant. Besides, mentioning the participant's selection criteria and including a brief description of the participants in the method section always adds transparency and rigor to the research.

## 4 Experiences and some points for consideration 3: participatory action research

The general notion of sampling is not appropriate terminology in the Participatory Action Research (PAR). In generic qualitative research, sampling refers to the participants who are “selected” by the researcher to receive the information and textual data during data collection. However, in PAR, the researcher who initiates the research at the beginning shares the ideas and invites people to contribute to the research process, and hence shares the responsibility and commitment to change in the research site, and they are collectively named ”co-researchers.“ In this article, I, the third author, share how co-researchers are invited and negotiate the roles and responsibilities of the PAR team. In a general sense, the popular and general method of sampling in qualitative research—purposive sampling (Campbell et al., 2020)—seems useful in PAR. However, the notion of purposive is rich and multilayered and needs further clarification in PAR.

Chevalier and Buckles (2019) mentioned that the researcher should first know the actors in the research process. In this regard, knowing the stakeholders who can potentially be co-researchers in a complex task is crucial. In this regard, the authors themselves raised some questions.

How can we focus on actors if we don't know what brings them together in the first place—i.e., the problems or goals they share? Likewise, how can we say that something is a problem without first discussing who decides what is the problem and what goals are being thwarted? Lastly, how can we develop goals for the future if we have no sense of the whole vision and interests they reflect, and what are the current failures in achieving them? (p. 243).

Having raised the question, it was finally mentioned that, in the end, problems, actors, and options are inseparable. This is a powerful and authentic set of questions that leads the process of confirming the “participants” (i.e., co-researchers) in PAR. Livingston and Perkins (2018) suggested that the role of the (academic) researcher is to facilitate discussions and understanding among the participants through scoping conversations at first and support them in agreeing upon the specific methods of inquiry. Here, the method of inquiry refers to the entire process of engagement into the field not as a passive way of information provider but through an active engagement in the cycle and action and reflection during knowledge generation.

Another popular method in PAR is respondent-driven sampling (Heckathorn, 1997). This is applicable when the population is ”hidden“ when no sampling frame exists, and public acknowledgment of membership in the population is potentially threatening, but there are people in the hidden population who can contribute to some actions and knowledge generation approaches. This can be done by a chain-referrals system in which one person refers to the other, and finally, the academic researcher makes a group of co-researchers. The analysis contains proof that, while sampling, like most chain-referral samples, begins with an arbitrarily defined set of initial subjects, the composition of the ultimate sample is entirely independent of those initial subjects.

Milne (2016) indicated that the participatory nature of research conceives a problem-solving technique that often involves researchers and research participants working together to examine a problematic situation, actions, or issues. So, participants should be mentally and emotionally ready to tackle such situations. In PAR, sometimes, people misconceive that this is a rigorous process, and participants are considered co-researchers, so the quality data might come from academic participants. In this context, Mata-Codesal et al. (2020) mentioned that non-academic participants themselves come to recognize, reflect on, and express their experiences in a novel way that may not be captured in basic texts through a conventional research method. It can be possible when artistic and creative practices capture such rich narratives of the field while researching and engaging with wider audiences. In this context, while forming a group of participants, the researchers should be aware of those people who can contribute in a noble way to the community toward using the research process to seek transformation in the direction of social justice rather than gathering people from the perspective of contributions through cognitive engagement.

## 5 Experiences and some points for consideration 4: ethnographic study

Ethnographic studies are inherently context and culture-sensitive, necessitating careful consideration of various socio-cultural factors in selecting both the research sites in which to “hang around” (Walford, 2018, para. 7) and the research participants with whom to engage for a prolonged period. Ethnographers prefer “participant selection” to “sampling” or “recruitment of participants” to ensure that the individuals chosen for the study reflect the socio-cultural dynamics of the context being researched (Atkinson, 2011). This approach acknowledges the complexity of human societies and emphasizes the importance of understanding cultural intricacies while respecting and honoring the cultural contexts of the participants.

First of all, ethnographers select research sites that are justified by specific criteria, as the site significantly influences participant selection. Once the research site is identified (of course, which should also be rationally justified with some specific criteria), the task of participant selection begins. Sometimes, the participants are clearly laid down in the research questions (e.g., Mayor, Minister, Secretary or Officer of the District/local education office, headteacher, School Management Committee Chair, or specific grade or subject teacher, or the students with a disability of a certain kind), in which case, the job becomes slightly easier—however, given the participants' right to not participate, we need to be open about moving on to another site. Again, the choice of site followed by participants may also not always be linear; it may be the other way round in some cases—after all, participants are the key focus. Therefore, researchers must remain flexible, as the choice of site and participants can be iterative rather than linear, with the potential to shift focus based on emerging insights during the research process (Nag, 2023). Moreover, detailed socio-cultural, economic, political, or other local dynamics of the site and participants are to be carefully and minutely considered in an ethnographic study to delve deeply into the intricacies of culture. The culture here means either or both community culture or institutional/group behavior.

While there is no watertight answer to how many participants would make an ideal ethnographic study, Angrosino's (2007) suggestion may be worthwhile—“the size of a sample depends on the characteristics of the group you are studying, on your own resources (i.e. legitimate limitations on your time, mobility, access to equipment, and so forth), and on the objectives of your study” (p. 48). For pragmatic reasons, the ethnographic practice in academic research has revealed that the number of participants in ethnographic studies typically ranges from six to ten, particularly when focusing on in-depth interviews and observations (Dhakal, 2021). This small size is conducive to generating rich qualitative data while allowing researchers to delve deeply into participants' experiences and interactions within their social contexts. If focus groups are included, the number may increase slightly. Importantly, the selection of participants is not merely about quantity; it is about achieving theoretical saturation, assuring that the data collected is comprehensive enough to address the research questions effectively. Therefore, it is the researchers' job to claim 'data saturation' and thus to limit the number of participants. In terms of our co-author's (Dhakal's) own experience and practice of engaging in ethnographic research and ethnographic sub-studies, he had tried to select the participants to best reflect the cultural or social groups being studied, especially focusing on small, purposefully chosen information-rich participants. The actual participants to interview/interact with or have group interactions (such as focused group discussion) may not be of a focus since most ethnographic studies rely on multiple methods of data generation—some of which do not require one-to-one or group interaction but only observations. Since ethnographers focus on observing daily interactions and participating in cultural activities, actual interviews with community members to gather narratives and stories may be relatively smaller.

Ethnographers employ various techniques of participant selection tailored to their research goals, such as purposive, snowball, convenience, or theoretical sampling. However, the purposive selection of participants is most commonly used in ethnographic research, as it facilitates the identification of information-rich participants who can provide deeper insights into the cultural dynamics being studied. While representation of diverse cultural or social groups is important, it is not the primary aim of ethnographic research. Instead, the focus is on selecting participants who can provide in-depth insights into the norms and behaviors of the community. And “the community” need not be regarded as homogenous. Different participants in the community may offer different accounts of what is valued. Critical ethnography also examines power relations and how these impact social interactions. So, a strong rationale must be clearly laid out for how and why the chosen number of participants would suffice and why they are the best-fit participants. Moreover, a detailed description of each participant's profile can be presented either as a summary table (see example Table 1) that I, the fourth author, had used in my PhD thesis on Women's participation in School governance' or detailed bio-sketch of each participant (see Table 1 from Dhakal's PhD). I had combined both the approaches in my PhD.

**Table 1:** Participant demographics.

**S.N**.	**Participants**	**Designation**	**Profile**
1	Milan	SMC Chair	Brahmin, man, early 60s, retired headteacher, educated, economically elite, earlier lived 1 km away from school, now lives 20 km away from school
2	Rajan	Headteacher	Brahmin, man, early 40s; educated, has good public relations, lives 500 m away from school
3	Nirmala	SMC Member	Brahmin, woman, mid-80s, economically and educationally disadvantaged, lives 500 meters away from school
4	Tika	SMC Member	Gurung, woman, early 30s; educated, economically elite, lives 3 km away from school
5	Sharmila	SMC Member	Sanyasi (calls her Kshatriya), woman, late 30s; literate, economically disadvantaged, prefers not to speak much; lives 2 km away from school
6	Bibek	SMC Member	Newar, man, mid-40s; educated, economically elite, local vendor/contractor, lives 500 meters away from school
7	Prabhakar	Political Activist (SMC Member)	Gurung, man, early 40s; educated, economically elite, entrepreneur, political cadre (turned leader, become ward chair after 2017 election) lives 500 m away from school

Source: (Dhakal, 2021, p. 67).

Table 1 shows the ways of presenting the participants' details aid in the transparency of the research process and participation selection procedure (Dhakal, 2021, p. 67).

## 6 Experiences and some points for consideration 5: case study

Qualitative case study is a popular research method in social science and educational research (Patnaik and Pandey, 2019). A case study can be defined as an in-depth examination of a complex subject(s), institution(s), problem(s), or subjects in a real-life setting (Crowe et al., 2011). The method is appropriate when the research question is “how” or “why” and the phenomenon is to be studied in a real-life or natural context (Grauer, 2012). There are two major types of case study research in terms of selection of case(s): single case and multiple. The subject of investigation in a single case study can be an individual, a family, a household, a community, an organization, an event, or even a decision (De Vaus, 2001), whereas in multiple case studies two or more cases can be studied. In this section, I, the fifth author, discuss participants' selection strategies in case studies to collect rich data for a holistic understanding of the studied phenomenon or phenomena.

The case study can be explanatory, exploratory, or descriptive. So, the case study design selection depends on the study's overall purpose (Yin, 2009). An explanatory case study seeks to identify the causal factors that explain a particular case. The primary focus of such a study is to explain “why” and “how” certain conditions come into being and why certain consequences of events occur or do not occur (Yin, 2014). An exploratory case study explores the context of the phenomenon, and its primary purpose is to investigate or identify the new research question that can be used extensively in succeeding research studies. Likewise, the primary purpose of a descriptive case study is to describe a phenomenon in detail in its real-life situation in which it occurred (Yin, 2009). In terms of the number of cases, it can be single and multiple case study research in which the researcher tries to have a holistic understanding of a unique, extreme, or critical case in a single case study, whereas, in contrast, the researcher explores the similarities or differences in multiple case studies. Sampling in a case study involves selecting representatives from a larger population for an in-depth analysis of the issue to be studied (Patnaik and Pandey, 2019). Qualitative case studies employ purposive sampling to illustrate the phenomenon of interest and present an in-depth understanding of the case of the study. In case study, like other qualitative research, participants are selected in terms of their relevance to the research topic or question(s) (Ishak and Baker, 2014).

Like other qualitative studies, even in case study research, researchers need to define participant selection criteria clearly and should have a specific purpose behind selecting the case(s), that offers valuable insights into the phenomenon under investigation. Similarly, considering the access and feasibility of data regarding availability, willingness to participate, the practicality of data collection methods, and continuity of the data collection process until it reaches the saturation point is also critical for determining samples. Moreover, the case study researchers select their cases gradually, not limiting the number of participants chosen until the data reaches saturation. Regarding this, Glesne and Peshkin (1992) suggest that if the stories are repeated among the participants and no new information is added to the research by any new participants, researchers need to stop selecting new participants.

For the sound, undulated, and unbiased study of the phenomenon under study, a case study involves multiple sources of data collection, like participant/nonparticipant observation, in-depth interviews, audio/video recordings, field notes, focus group discussion (FGD), conversations in a natural setting, and study of documents (whether of books, archival manuscripts, signs, physical artifacts, and so on) (Priya, 2020). The concept of conducting an “unbiased” study is highly debated, even within qualitative research (Guba and Lincoln, 1989). In constructivist credo, Lincoln and Guba (2016) noted that researchers do not need to assert their impartiality but should instead embrace a dialogical approach with their participants or co-researchers. Gergen (2014) further emphasizes that achieving excellence in qualitative research is less about striving for objectivity and more about fostering strong relationships with research participants. The multiple sources of data collection are crucial in the case study, which does not seek to offer a more or less unbiased representation, but it can be used to enhance dialogically generated insights and increase the richness and quality of the findings, which is likely to be more convincing and accurate (Patnaik and Pandey, 2019). At the same time, due to the bulk of data from multiple sources, sometimes there is the risk of the researchers being lost in the data. Romm (2018) expanded the discussion to provide more detailed accounts of what acting responsibly toward research participants means. Therefore, Baxter and Jack (2008) suggest proper organization and analysis of the data as each data source is one piece of the “puzzle,” each contributes to the researcher's holistic understanding of the phenomenon. Thus, the emphasis is not solely on the professional researcher's comprehension but on co-creating understanding and insight (Lincoln and Guba, 2016; Gergen, 2014).

## 7 Experiences and some points for consideration 6: grounded theory

Grounded theory is a highly favored qualitative research methodology in the social sciences because of its distinct theory development process. In contrast to conventional research methods, Grounded Theory enables theories to arise directly from methodically gathered and examined data (Liu, 2022). Studying social interactions, processes, and behaviors in natural environments is where this approach works. This approach emphasizes the development of theory from the bottom up, which gives researchers a more authentic understanding of the phenomenon they are studying and helps them gain insights into the lived experiences of participants (Noble and Mitchell, 2016). In this sense, grounded theory has proven to be an effective tool for examining complicated social issues, especially poorly understood ones, because of its versatility and flexibility.

As grounded theory aims to build a theory grounded in the data, participant selection is crucial. Grounded theory adopts theoretical sampling as an effective strategy as it is a dynamic, iterative process that is driven by emerging theory (Cho and Lee, 2022). Researchers continuously gather and analyze data using this sampling method by letting the developing theory determine where, when, and from whom further data should be collected (Conlon et al., 2023). As a result, this method enables researchers to concentrate on their areas of interest, find theoretical gaps as they occur, and ensure that the final theory is thorough and solidly supported by the available data.

A broad research question or area of interest is usually the starting point for theoretical sampling. The initial data collection process may involve document analysis, observations, or interviews, depending on the research context. Furthermore, the emerging theory guides the sampling decisions as they are gathered and analyzed. In this regard, the researcher looks for more subjects or data sources that can elaborate on that idea (Da Silva Barreto et al., 2023). Ensuring that the final theory is thorough and firmly based on the data enables the researcher to expand and improve the theory as the study goes on. Another element to consider during the grounded theory sampling process is reflexivity. In qualitative research, reflexivity is crucial, especially when using techniques like grounded theory, where the researcher's work is closely linked to gathering and analyzing data (Draucker et al., 2007). By practicing reflexivity, researchers become conscious of their own prejudices and how they might affect the way they conduct their work, including how they choose to sample. Neill (2006) emphasized the importance of reflexivity to ensure that researchers' preconceptions do not influence the sampling process, keeping it aligned with the emerging theory's requirements. Charmaz (2015) argued that grounded theory can never be a completely objective representation of phenomena. Researchers should transparently disclose how their theories have been constructed or co-constructed (Mills et al., 2006). Mills et al. (2006) offer a comprehensive discussion of the nuances within grounded theory and constructivist approaches, including those explicitly promoted by Charmaz (2015) and other proponents. By practicing reflexivity, researchers can address potential biases, enhancing the quality and credibility of their data collection. This process strengthens the rigor of grounded theory and the research relationship.

The process of theoretical sampling is a continuous cycle of data collection, analysis, and refinement rather than a linear one. To find trends, concepts, and categories, newly acquired data is instantly examined and contrasted with the database. A key component of grounded theory, constant comparison, ensures that the evidence supports the developing theory (Strauss and Corbin, 1993). The process of theoretical sampling persists until theoretical saturation is achieved, which transpires when supplementary data ceases to advance the theory (Charmaz, 2022, p. 92). At this stage, the researcher can be sure that the theory appropriately explains the phenomena being studied and is well-supported by the data.

According to Thomson (2010), a sample size of about 25 interviews is typical for grounded theory research. A larger sample size of up to 30 interviews might be advised in some circumstances, though, to thoroughly develop the patterns, concepts, categories, attributes, and dimensions of the phenomenon being studied. Researchers can examine the subtleties of the data and make sure the developing theory is thorough and well-supported with a larger sample size.

Grounded theory carefully considers sample diversity in addition to sample size. Depending on what the study requires, the sample consists of people with a range of experiences, backgrounds, and viewpoints (Cho and Lee, 2022). Researchers can make sure that the developing theory accurately reflects the complexity of the phenomena they are studying by enlisting the assistance of a wide range of participants. However, the idea of accuracy is challenged by constructivist grounded theorists—the developing theory is a co-construction that can never be checked for accuracy against some objective reality. So, it is deemed to offer insights in relation to the complexity of the phenomena being researched.

Likewise, another important component of grounded theory is the consideration of ethical issues. The requirements of the developing theory dictate the sampling procedure. Because of this, researchers need to consider any potential ethical ramifications before making any sampling decisions. Researchers should take into account concerns about participant impact, informed consent, and confidentiality, according to Conlon et al. (2023). By doing this, researchers can ensure that participants are treated fairly and respectfully during sampling. The rigor and credibility of the research are enhanced by important aspects of sampling in grounded theory, including reflexivity, sample size, diversity, and ethical considerations.

## 8 Experiences and some points for consideration 7: phenomenology

Phenomenology is a unique qualitative form of inquiry into lived experiences of human existence, and it aims to understand those experiences from the participants' perspectives (van Manen, 2007, 2017). This methodology is ingrained in early 20th-century European philosophy, which comprises the use of thick descriptions of close inquiry of lived experience to understand how meaning is created through personified insights and perceptions (Sokolowski, 2000). Digging deep into participants' lived experiences that reflect their life's pains and gains is a challenging job for a researcher. For instance, “a study on the lived experiences of pregnant women with psychosocial support from primary care midwives will recruit pregnant women varying in age, parity and educational level in primary midwifery practices” (Moser and Korstjens, 2018, p. 11). It is essential to appropriately select research phenomena and participants and formulate research questions for a phenomenological study (Barton, 2020; Duffy and Mhuirthile, 2024) to capture the essence of the participants' shared experience and construct meaning from their experiences.

The term “sample within phenomenological methodology should not refer to an empirical sample as a subset of a population”, but to a wisely chosen group of human beings that share in-depth insights into the essence of the subject being studied (van Manen, 2016, p. 352) aligned to transformative intents. Phenomenological researchers primarily employ purposive, snowball, and maximum variation strategies for selecting their research participants (Creswell, 2007; Maxwell, 2013; Mertens, 2010). These strategies help researchers delve deep into their participants' lived experiences about the phenomenon being studied. Purposive sampling is a key data collection strategy in phenomenological study as it enables researchers to select participants with a rich array of lived experiences of the phenomenon under study and willing to provide rich, thorough, and evocative data (Palinkas et al., 2015). The participants are selected based on their lived experiences, knowledge of the phenomenon being studied, and their verbal efficiency in describing their group or (sub)culture (van Manen, 2016). The participants can provide rich descriptions of their experiences with the phenomenon, collaborating with the researcher to explore its essence and construct meaning.

Another important information-gathering strategy is snowballing. While selecting research participants through snowball sampling, the researcher first selects one or a few participants, considering their knowledge and ability to express their experiences about the phenomenon under study. Then, they recognize other prospective participants who are supposed to have in-depth information about the phenomenon, for example, a program or community, being explored by requesting initial participants to recommend other people who possess similar characteristics and experiences (Creswell, 2007; Mertens, 2010; Palinkas et al., 2015). Mertens (2010) further states that the list of participants grows, like a snowball, as the added participants refer to other prospective members' names. Hence, the initial participants' recommendations help a phenomenological researcher conveniently select the appropriate study participants.

Maximum variation sampling is another significant strategy to gather intentionally heterogeneous data for phenomenological research (Robinson, 2014). The researcher selects participants with a wide range of characteristics and experiences about the phenomenon being explored. The data collected from such a selection of participants can yield varied information from a wide range of perspectives and identify important common patterns (Creswell, 2007). The participants share experiences of a phenomenon being explored in a phenomenological study (Creswell, 2013). Phenomenological study focuses on gathering the depth and quality of the information primarily through interviews and observations rather than the number of participants.

There are different opinions regarding the number of participants in qualitative research, including phenomenology. For example, Polkinghorne (1989) suggests interviewing 10 to 25 participants, and Moustakas (1994) recommends that a researcher should take between 5 and 25 participants. However, the gathering of data continues until saturation occurs or when the data no longer reveals new insights or themes from the participants (Creswell, 2013). The researcher encourages and probes their participants to describe their experiences in detail during the unstructured interviews and semi-structured interviews and observes them in the context where the phenomenon being explored is experienced (Starks and Trinidad, 2007). A researcher can use an unstructured interview if they have a limited understanding of the topic and want to rely on their participants' information to lead the conversation and a semi-structured interview in order to obtain in-depth data from the participants (Parish and Shaikh, 2023). The data is expected to reach a point of saturation from the participants, ensuring that no new understandings or themes would emerge from further participants (Morse, 2000). Saturation occurs when the data no longer reveals new information or themes, and further interviews or data collection yields redundant information (Creswell, 2013).

The primary emphasis of a phenomenological study is on the richness and saturation of the information, so selecting appropriate participants is a critical methodological process. A researcher can obtain in-depth, diverse, and evocative insights into participants' lived experiences by employing purposive, snowball, or maximum variation sampling strategies, ensuring that the selection process benefits both researchers and participants. The number of participants is decided when the insights or themes start getting repeated, affirming the study encapsulates the essence of the phenomenon being explored. Hence, an appropriate selection of participants helps researchers construct an in-depth understanding of human experiences based on the viewpoints of those who have experienced them.

## 9 Closing thoughts

Participant selection is a critical aspect of qualitative research designs that influences the credibility and richness of the data collected (Dahal, 2023). This article has provided a comprehensive guide and offers some thinking points for consideration for novice and/or veteran researchers, drawing from the authors' extensive experiences in different qualitative methodologies, including auto/ethnography, narrative inquiry, participatory action research, ethnography, case study, phenomenology, and grounded theory.

The nuanced differences and unique aspects of participant selection across these methodologies (as shown in Table 2) highlight the importance of a thoughtful and deliberate approach. However, considering the problem, purpose, research question, and theoretical framework, researchers can ensure that their participant selection process aligns with the goals of their study and enhances the overall quality of their research without compromising their methodology. This article also emphasized the iterative nature of qualitative research design, such as auto/ethnography, where participant selection is not a one-time decision in qualitative research design but an ongoing process that may require adjustments as the study progresses as an emerging nature of the qualitative inquiry. Ethical considerations, our experiences, and some thinking points in this serve as valuable guidelines for researchers to navigate the complexities of participant selection.

**Table 2:** Overview of participant selection across qualitative methodologies.

**Method(ology)**	**Key features of participant selection**	**Sample size**	**Key considerations**
Auto/ethnography	Focus on personal and cultural narratives, exploring self and others' experiences; iterative process.	Varies (context-dependent)	Ethical considerations; prolonged engagement with participants; evolving selection during research.
Narrative inquiry	Minimal participants, emphasizes temporality, sociality, and place	Minimum 4–6 (depends on focus)	Selection criteria are based on research purpose, theoretical framework, and rich and meaningful narratives.
Participatory Action Research (PAR)	Collaborative selection of participants as co-researchers emphasizes shared ownership	Flexible	Involves stakeholders with shared goals; purposive and respondent-driven sampling; empowerment of non-academic participants.
Ethnography	Focus on socio-cultural context; participants reflect cultural dynamics; iterative process	6–10	Selection is based on the research site; purposive sampling is based on the research site, purposive sampling, and profiles or bio-sketches to enhance transparency.
Case study	Guided by research questions; purposive sampling for relevance; includes single or multiple cases	Until data saturation	Clear selection criteria; triangulation of data sources; manage data overload to maintain focus.
Grounded theory	Theoretical sampling guided by emerging theory; an iterative process	25–30 interviews	Diversity in the sample, constant comparison and theoretical saturation; reflexivity to avoid biases.
Phenomenology	Focus on lived experiences; uses purposive, snowball, and maximum variation sampling	5–25 participants	Thick descriptions from participants; saturation ensures no new themes; primary data from interviews and observations.

Starting with the participant selection procedure in auto/ethnographic inquiry, the first author discusses and further exemplifies the adaptability and depth of qualitative research. This approach blends personal and cultural narratives, allowing researchers to connect individual stories to broader social and cultural contexts. The selection of participants in auto/ethnography is also guided by the research purpose and the need to explore both self and others' experiences in a meaningful way. However, narrative and auto/ethnographic inquiries underscore the iterative nature of qualitative research, where participant selection is an ongoing process that may evolve as the study progresses. Ethical considerations and the need for prolonged engagement with participants are essential to ensuring the richness and credibility of the data collected.

Next, the narrative inquiry that emphasizes the importance of temporality, sociality, and place involves a minimal number of participants, sometimes even just one, especially in autobiographical or life history approaches. The selection criteria are typically based on the research purpose, problem, and theoretical framework, ensuring that the participants' stories are rich and meaningful. For instance, in the second author's PhD study, the selection of experienced English language teachers in Nepal was based on specific criteria to ensure depth and relevance in the narratives collected.

Further, by subscribing to participatory action research (PAR), the third author redefines the traditional sampling concept by emphasizing the research process's collaborative nature. In contrast to conventional qualitative research, where the researcher chooses the participants, PAR entails inviting people to join as co-researchers who share responsibility and dedication to the research objectives. This approach fosters a sense of ownership and active engagement among all participants, enhancing the relevance and impact of the research. The process of identifying co-researchers in PAR is complex and multifaceted. It requires understanding of the stakeholders, their shared problems or goals, and the broader vision they aim to achieve. This collaborative approach ensures that the research is grounded in the real-world experiences and aspirations of the community involved. Methods such as purposive sampling and respondent-driven sampling can be adapted to fit the unique needs of PAR, ensuring that the co-researchers are well-suited to contribute meaningfully to the research process. The participatory nature of PAR encourages a problem-solving mindset, where researchers and co-researchers work together to address issues and generate knowledge. And this idea of collaboration (and co-generation of meaningful insights) is not confined to PAR. It can enter much qualitative research (Gergen, 2014). This collaborative effort often leads to richer, more nuanced data that might be captured through something other than traditional research methods. The inclusion of non-academic participants who bring diverse perspectives and experiences further enriches the research outcomes. PAR transforms the research process into a collective journey of inquiry and action, where the roles and responsibilities are shared, and the knowledge generated is co-created. This approach not only enhances the quality and relevance of the research but also empowers the participants, fostering a deeper connection to the research outcomes and their potential impact on the community. Researchers can create more inclusive, impactful, and ethically sound research practices by embracing the principles of PAR.

Likewise, the fourth author added that ethnographic studies require a deep understanding of the socio-cultural context and careful consideration of participant selection. Unlike other qualitative methodologies, ethnography emphasizes “participant selection” oversampling to ensure that the chosen individuals reflect the research site's cultural dynamics and enable (critical) exploration of power relations. This approach respects the complexity of human societies and honors the cultural contexts of the participants. The process begins with selecting a research site based on specific criteria, which significantly influences participant selection. Flexibility is crucial, as the choice of site and participants can be iterative, adapting to emerging insights during the research process. Detailed consideration of socio-cultural, economic, and political dynamics is essential to delve deeply into the intricacies of the culture being studied. The number of participants in ethnographic studies typically ranges from six to ten, focusing on in-depth interviews and observations. This small size allows for rich qualitative data while ensuring comprehensive coverage of the research questions. Achieving theoretical saturation is key, ensuring that the data collected is sufficient to address the research objectives effectively. Ethnographers employ various participant selection techniques, with purposive sampling being the most common. This method identifies information-rich participants who can provide deep insights into the cultural norms and behaviors of the community. The goal is to represent diverse groups and select participants who can contribute meaningfully to the study. Providing detailed profiles of participants enhances the transparency of the research process. This can be done through summary tables or detailed bio-sketches, offering a clear rationale for the selection and demonstrating how the participants' characteristics align with the research goals. Ultimately, ethnographic research is about understanding and interpreting the lived experiences of individuals within their cultural contexts. By carefully selecting participants and considering the socio-cultural dynamics, researchers can produce rich, nuanced insights that contribute to a deeper understanding of human societies.

Next, in qualitative case study research, the fifth author reported that qualitative case study offers a robust method for exploring complex phenomena within their real-life contexts. This approach is particularly valuable in social science and educational research, where understanding the intricacies of specific cases can provide deep insights into broader issues. Case studies can be explanatory, exploratory, or descriptive, each serving different research purposes. The selection of cases, whether single or multiple, is guided by the research questions and the need to understand the phenomenon in depth. Also, it is important to consider who is setting the research questions so that the research can benefit participants, especially those most marginalized in the social fabric (and, for that matter, the ecological fabric). Single case studies focus on unique or critical cases, while multiple case studies explore similarities and differences across several cases. Participant selection in case studies is a critical process that involves purposive sampling to ensure that the participants are relevant to the research questions. Defining clear selection criteria and considering factors such as availability, willingness to participate, and data collection feasibility are essential. The goal is to continue selecting participants until data saturation is achieved, ensuring additional participants add no new information. The richness of case study research lies in its use of multiple data sources, including observations, interviews, recordings, field notes, focus group discussions, and document analysis. This triangulation of data sources enhances the credibility and depth of the findings. However, researchers must be cautious of the potential for data overload and ensure proper organization and analysis to maintain a clear focus on the research objectives. Thus, qualitative case study research provides a comprehensive and nuanced understanding of the studied phenomena. By carefully selecting participants and employing multiple data collection methods, researchers can produce convincing and accurate findings, contributing valuable insights to the field.

All the above, the sixth author added that grounded theory stands out as a powerful qualitative research methodology, particularly valued for its ability to generate theories directly from systematically gathered and analyzed data. This bottom-up approach provides researchers with authentic insights into social interactions, processes, and behaviors within their natural environments, making it especially effective for exploring complex and poorly understood social issues. Participant selection in grounded theory is a dynamic and iterative process known as theoretical sampling. This method allows the emerging theory to guide the selection of participants, ensuring that data collection is continuously refined and focused on filling theoretical gaps. This iterative process, combined with constant comparison, ensures that the developing theory is robust and well-supported by the data. Reflexivity is another crucial element in grounded theory, helping researchers remain aware of their biases and ensuring that these do not influence the sampling process. By practicing reflexivity, researchers can enhance the quality and credibility of their data collection, thereby strengthening the overall research process. Theoretical sampling continues until theoretical saturation is achieved, meaning no new data significantly advances the theory. This ensures that the final theory is comprehensive and accurately reflects the phenomena being studied. While a typical sample size for grounded theory research is around 25 interviews, it may extend to 30 to develop theoretical constructs thoroughly. Diversity in the sample is also essential, as it ensures that the emerging theory captures the complexity of the phenomena under study. Researchers must consider a range of experiences, backgrounds, and perspectives to develop a well-rounded theory. Ethical considerations are paramount in grounded theory research. Researchers must ensure that participants are treated with respect and fairness, addressing issues such as informed consent, confidentiality, and the potential impact on participants. Researchers must examine the likely consequences with participants and ways of setting research questions and proceeding with the research. By adhering to these ethical standards, researchers can enhance the rigor and credibility of their studies. Overall, grounded theory provides a flexible and rigorous framework for developing theories that are deeply rooted in empirical data. By carefully considering participant selection, reflexivity, sample diversity, and ethical issues, researchers can produce robust and meaningful theories that contribute significantly to our understanding of complex social phenomena.

Finally, the seventh author adds that phenomenology offers a thoughtful approach to understanding the lived experiences of individuals, focusing on capturing the essence of these experiences from the participants' perspectives. Rooted in early 20th-century European philosophy, phenomenology employs thick descriptions and close inquiry to uncover how meaning is constructed through personal insights and perceptions. Selecting participants for phenomenological study is a critical process that goes beyond traditional sampling methods. Instead, it involves purposive, snowball, and maximum variation strategies to ensure that participants provide rich, detailed accounts of their experiences. Purposive sampling allows researchers to choose individuals who have deep insights into the phenomenon, while snowball sampling helps identify additional participants through recommendations from initial subjects. Maximum variation sampling ensures a diverse range of perspectives, enhancing the depth and breadth of the data collected. The number of participants in phenomenological research varies, with recommendations ranging from 5 to 25 participants. The key is to continue data collection until saturation is reached, meaning no new themes or insights emerge from additional data. This ensures that the study captures the full essence of the phenomenon being explored. Interviews, both unstructured and semi-structured, are primary data collection methods in phenomenology. These interviews allow participants to describe their experiences in detail, providing the rich, evocative data needed to understand the phenomenon thoroughly. Observations in the context where the phenomenon occur further enrich the data. The success of a phenomenological study hinges on the careful selection of participants and the depth of the data collected. By employing appropriate sampling strategies and focusing on the richness and saturation of the information, researchers can construct a comprehensive understanding of human experiences grounded in the authentic perspectives of those who have lived them. These days, many qualitative researchers point out that the construction should not lie in the hands of professional researchers but must be a co-construction with participants, with the intent that the research will benefit (marginalized) participants. This approach not only enhances the credibility and depth of the research but also provides valuable insights into the complexities of human existence.

In summary, Table 2 shows the overview of participant selection across selected qualitative methodologies, and Table 3 shows participant selection considerations by methodologies.

**Table 3:** Participant selection considerations by methodologies.

**Method(ology)**	**Purpose**	**Sampling method**	**Ethical and practical aspects**
Auto/ethnography	Explore self and cultural narratives	Purposive, evolving	Respect participant autonomy; ensure cultural and contextual depth.
Narrative inquiry	Understand individual stories and broader meanings	Purposive, criterion-based selection	Respect participants' stories; engage deeply over time.
PAR	Foster co-research and community action	Purposive, collaborative	Ensure inclusivity; address power dynamics; foster mutual respect.
Ethnography	Analyze cultural norms and behaviors	Purposive, site-based	Ensure participants reflect cultural diversity; maintain prolonged engagement.
Case Study	Explore real-life phenomena in depth	Purposive, saturation-focused	Obtain informed consent; ensure data quality across multiple sources.
Grounded Theory	Develop theory from data	Theoretical sampling	Reflexivity; diversify perspectives; balance emerging insights.
Phenomenology	Capture the essence of lived experiences	Purposive, snowball, maximum variation	Ensure rich, detailed accounts; maintain confidentiality.

In closing, this article shall empower novice and/or veteran, graduate, and postgraduate researchers with the knowledge and tools needed to make informed decisions about participant selection considering some thinking points, thereby contributing to the rigor and richness of qualitative research in educational contexts. With our experiences and insights, we hope to foster a deeper understanding of the participant selection process and inspire researchers to approach it with the care and consideration it deserves.

## Data Availability

The raw data supporting the conclusions of this article will be made available by the authors, without undue reservation.
